# Training Set Optimization for Sparse Phenotyping in Genomic Selection: A Conceptual Overview

**DOI:** 10.3389/fpls.2021.715910

**Published:** 2021-09-09

**Authors:** Julio Isidro y Sánchez, Deniz Akdemir

**Affiliations:** ^1^Centro de Biotecnologia y Genómica de Plantas, Instituto Nacional de Investigación y Tecnologia Agraria y Alimentaria, Universidad Politécnica de Madrid, Campus de Montegancedo, Madrid, Spain; ^2^Animal and Crop Science Division, Agriculture and Food Science Centre, University College Dublin, Dublin, Ireland

**Keywords:** training set optimization, genomic selection, genome-wide markers, statistical design, sparse phenotyping, genomic prediction, mixed models

## Abstract

Genomic selection (GS) is becoming an essential tool in breeding programs due to its role in increasing genetic gain per unit time. The design of the training set (TRS) in GS is one of the key steps in the implementation of GS in plant and animal breeding programs mainly because (i) TRS optimization is critical for the efficiency and effectiveness of GS, (ii) breeders test genotypes in multi-year and multi-location trials to select the best-performing ones. In this framework, TRS optimization can help to decrease the number of genotypes to be tested and, therefore, reduce phenotyping cost and time, and (iii) we can obtain better prediction accuracies from optimally selected TRS than an arbitrary TRS. Here, we concentrate the efforts on reviewing the lessons learned from TRS optimization studies and their impact on crop breeding and discuss important features for the success of TRS optimization under different scenarios. In this article, we review the lessons learned from training population optimization in plants and the major challenges associated with the optimization of GS including population size, the relationship between training and test set (TS), update of TRS, and the use of different packages and algorithms for TRS implementation in GS. Finally, we describe general guidelines to improving the rate of genetic improvement by maximizing the use of the TRS optimization in the GS framework.

## 1. Introduction

The rate of genetic gain in plant breeding must be enhanced to meet the demand of humanity for agricultural products in the next few decades (Xu et al., 2020). Tools, such as genomic assisted breeding (GAB), that improve the understanding of structural and functional aspects of plant genomes are key in modern breeding methods. GAB can be defined as the set of breeding tools (next-generation sequencing, omics information, and statistics) that study whole genomes by integrating multiple disciplines with new technology from informatics and robotic systems to improve selection and mating in plant breeding programs (Varshney et al., 2005, 2021). In GAB, other tools such as genetic transformation and genome editing are currently playing a key role to select better-adapted genotypes while pursuing faster genetic gains (Zhang et al., 2018). One of the emergent methodologies within GAB that have revolutionized plant and animal breeding is genomic selection (GS). GS is considered the most promising tool for genetic improvement of the complex traits controlled by many genes, each with minor effects because (i) GS can increase the rates of genetic gain through increased accuracy of estimated breeding values (Heffner et al., 2009), (ii) significantly shorter breeding cycles (Crossa et al., 2017), and (iii) the better utilization of available genetic resources through genome-guided mate selection (Akdemir and Sánchez, 2016).

Breeders test candidate genotypes in multi-year and multi-location trials to select superior genotypes with high performance. This approach limits the number of variety candidates to be tested, and it is the main cause of the fact that plant breeding programs are time and cost-intensive. A breeding tool that combines the power of GS and the potential of an extensive collection of germplasm, assisted by new technologies, will offer promise in crop breeding to contribute to global food security (Xu et al., 2020) because it can accelerate the generation interval by reducing the generation time in plant breeding programs (Falconer and Mackay, 1996).

Bernardo (1994) was the first who proposed the use of genomic information as covariates for predicting untested genotypes but it Meuwissen et al. (2001) who came through with a new methodology to deal with the challenge of fitting prediction models when the number of genomic covariates (markers, p) is larger than the number of data points (n). Since then, simulations and empirical studies have demonstrated that GS could greatly accelerate the breeding cycle (Heffner et al., 2009), maintain genetic diversity within the breeding programs, and increase genetic gain beyond what is possible with phenotypic selection or quantitative trait loci (QTL) mapping approaches (Crossa et al., 2017). Genomic selection is a breeding tool that uses supervised machine learning approach with a training set (TRS) to predict genomic estimated breeding values (GEBVs) of an un-phenotyped test set (TS). (Isidro et al., 2016) of genotypes. The prediction of GEBVs involves a whole-genome regression model in which the known phenotypes are regressed on the markers. The GS models are trained on data that consists of both phenotypic and genome-wide markers data that is used to estimate marker (or lines) effects de los Campos et al. (2013). The combination of the marker effect estimates and the marker data from the TS is used to calculate GEBVs for the TS. The selection of individuals is based on the GEBVs as the selection criterion. The performance of the GS model is determined by calculating the correlation between GEBVs (genomic predictions) and the unknown true breeding value. As the true breeding values are never known, the available phenotypic records in the TRS are used by cross-validation values to evaluate GS. This is called prediction ability and should not be confused with prediction accuracy. The latter provides an estimate of the genotypic correlation and is estimated as the prediction ability divided by the square root of the heritability for the trait being predicted (Dekkers, 2007; Lee et al., 2008; Lorenzana and Bernardo, 2009; Riedelsheimer et al., 2012). Enhancing GS accuracy is very important for the success of GS breeding programs since the expected genetic gain from GS is directly proportional to the accuracy of GS models (Crossa et al., 2010; de los Campos et al., 2013).

There are many factors affecting the accuracy in GS by interacting in a complex network relationship (Zhong et al., 2009; Isidro et al., 2016; Liu et al., 2018; Zhang et al., 2019). Within these factors, there is one that is key to the accuracy of the prediction models in GS, and it is the design of the TRS since the predictability of a model is critical for the success of GS. In this study, the aim is to shed some light on the different TRS optimization criteria by covering the fundamentals of TRS optimization and its uses in GS, including selection strategies for long-term gains. We focus on reviewing the TRS methods from the literature that can be used as tools for designing a TRS and constructed an example to compare the TRS optimization strategies.

## 2. Populations in GS

**Figure 1:**
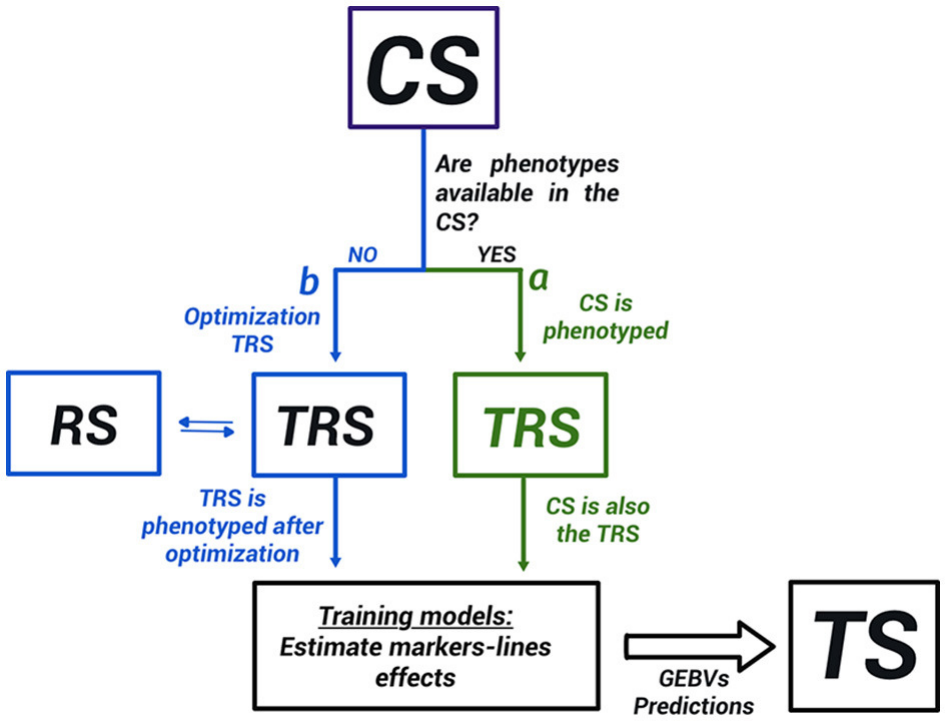
Populations in genomic selection (GS). CS, calibration set, TRS, training set, RS, remaining set, and TS is the test set. When CS has all the phenotypic and genotypic information, CS and TRS are the same populations. Otherwise, we could have up to four different populations in the GS scheme. There are two different types of TRS selection problems: in one of these (a), the CS is already phenotyped and genotyped, and a subset of the CS is used as a TRS in the modeling stage. In the other (b), the CS is only genotyped and a TRS is constructed by phenotyping a subset of the CS.

Genomic selection requires training of statistical models on available genotypic and phenotypic data from a TRS to make predictions about new genotypes. The selection of TRS involves different populations (Figure 1):

1. A calibration set (CS): is the group of genotypes available for the breeders from which the TRS is selected. If the individuals in this CS are phenotyped and genotyped, the populations for GS will be CS (TRS) and TS, and in theory, no need for optimization of the TRS (branch a in Figure 1). Nevertheless, a subset of the CS might be preferable, i.e., if very distant individuals (Lorenz and Smith, 2015) are present, to include or exclude extreme phenotypes (Lopez-Cruz and de Los Campos, 2021), or to remove irrelevant individuals (Brandariz and Bernardo, 2018). If only genotypic information is available and just a subset of them can be used for phenotyping due to budget restrictions, then a TRS will be carefully identified from the CS (branch b in Figure 1).
2. Training set (TRS): is where the prediction equation will be built. The TRS individuals present genotypic and phenotypic information. Under budget constraints, the aim is to select the minimum number of genotypes to phenotype, but that will assure an optimal accuracy on the TS population. The selection of the best genotypes to select from the CS to create the TRS is called optimization of the TRS. In TRS, the true response values are known (phenotypes). In this study, we used both the genotype and phenotype information from the TRS to obtain a prediction equation, which predicts the effect of each marker (or line) on the trait.
3. Remaining set population (RS): is the remaining genotypes in the CS that are used in the process of optimization. It could be also reserved for evaluating the performance of the statistical model before making predictions if the phenotypic information is available.
4. Test or Target set (TS): is the set of genotypes to predict. Only genotypic information is available in this population.

Therefore, the different populations in GS depend on whether or not the phenotypic information is available within the CS. Figure 1 shows the distinction between the two major groups of TRS optimization methods found in the literature. The first group of methods addresses the situation where the phenotypic information is already available in the CS (Neyhart et al., 2017; Brandariz and Bernardo, 2018; Lopez-Cruz and de Los Campos, 2021). They aim to use only a part of the CS when building a GS model excluding irrelevant genotypic and phenotypic information. For instance, constructing a TRS from only the individuals with high or low values of the phenotypes (Neyhart et al., 2017; Brandariz and Bernardo, 2018), or the more recently proposed sparse modeling approach Lopez-Cruz and de Los Campos (2021). The second group of methods, which is the main focus of discussion in this study, assumes that the phenotypic information is not available in the CS, and will be obtained after selecting a TRS. In this case, the resources of the breeding program are limited and just a subset of the individuals can be phenotype. In this situation, the TRS must be carefully built within the CS through an optimization process, and distinguish four different populations (CS, TRS, RS, and TS; Figure 1). In both groups of methods, the model validation is usually accomplished by cross-validation within the TRS (Heffner et al., 2009; Luan et al., 2009).

In general, within the TRS optimization framework, when the objective is to select a TRS to predict the remaining individuals from the same population we talk about *Un-targeted TRS*. Likewise, when a TS is first defined and genotyped, and then the TRS is optimized specifically around the TS then we define a *targeted TRS*. It is important to note, that not all optimization criteria are sensitive to this distinction, (i.e., refer next section, PAM, A-OPT, D-OPT), nevertheless, when it is so, this is reflected in how the optimization criteria are calculated (Lorenz and Smith, 2015; Akdemir and Isidro-Sánchez, 2019).

In addition, when there is heterogeneity within the environment such as row/column effects in the field, the optimal TRS of the phenotypic experiment involves not only the selection of the TRS but also the placement of genotypes in the environment (Heslot and Feoktistov, 2020). The experimental design might need blocking structure and environmental covariates and in these cases, the order in which the individuals are positioned in the environment will be important. We refer to this kind of optimization as the "ordered" optimization as opposed to the “unordered” optimization (Akdemir et al., 2021).

## 3. Design Optimization Criteria

The TRS optimization process is an optimal experimental design problem, and many aspects of GS implementation captured the attention of statisticians in the past (Smith, 1918; Kiefer, 1959; Fisher, 1960; Fedorov, 1972; Atkinson and Donev, 1992; Pukelsheim and Rosenberger, 1993; Fedorov and Hackl, 2012; Silvey, 2013). The design of the concept of the experiment should be more used to plan experimental designs in plant breeding programs and perform sets of well-selected optimization TRS to get the most informative combination out of the given factors.

The most common design optimization criteria method is indisputably the classical simple random or stratified sampling, mainly because of its simplicity and generality (Gentle, 2006), but also because of the difficulty to sample more efficiently when the number of candidate solutions is large. We classified the different design optimization criteria in to three major groups.

**Figure 2:**
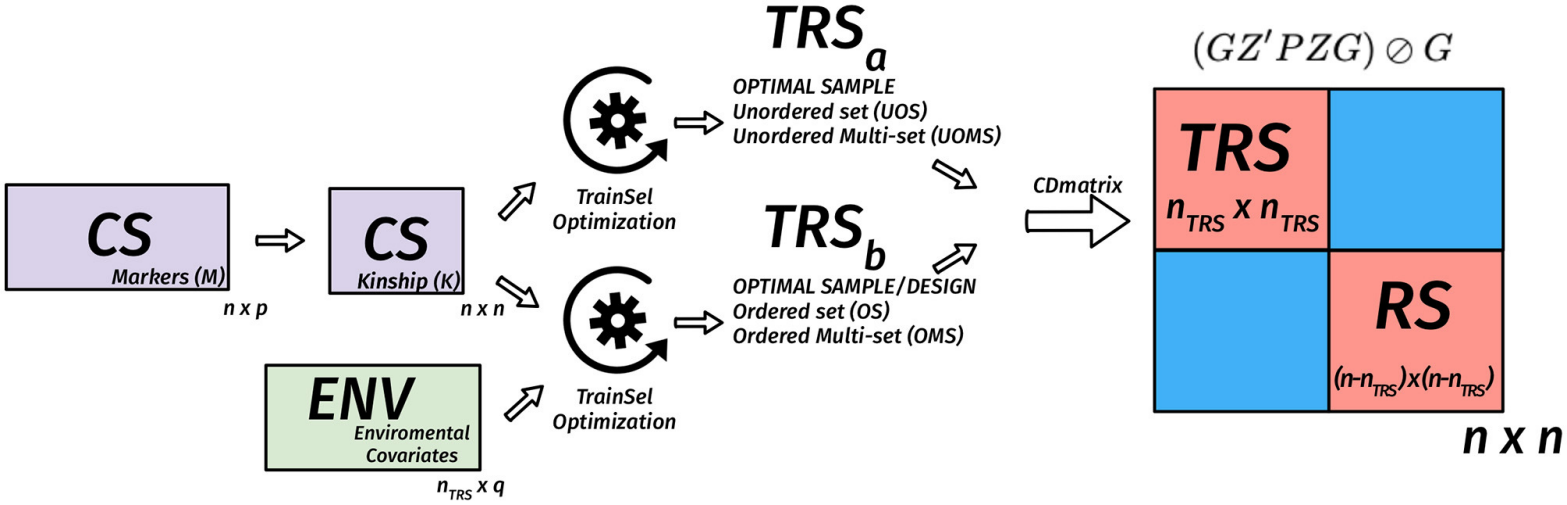
Diagram of the matrix of the coefficient of determination (CD) criterion in the TRS optimization for unordered and ordered experiments for the un-targeted case. We assume that we have n genotypes in the candidate set, *n*_*TRS*_ genotypes are selected to the TRS. In this case, the *n* × *n* kinship matrix is used to calculate the *n* × *n* CD matrix for a given TRS, then the mean CD is calculated based on certain diagonals that correspond to TRS or remaining set (RS). The optimization algorithm is used to find the best TRS. When provided with a design matrix that has heterogeneous rows, then we are also looking for a design in addition to the selection of a TRS. In this case, both the kinship matrix and the *n*_*TRS*_ × *q* environmental covariates matrix are used to calculate the *n* × *n* CD matrix for a given TRS, then the mean CD is calculated based on certain diagonals that correspond to TRS or RS. The optimization algorithm is used to find the best TRS and best design with this TRS.

**Figure 3:**
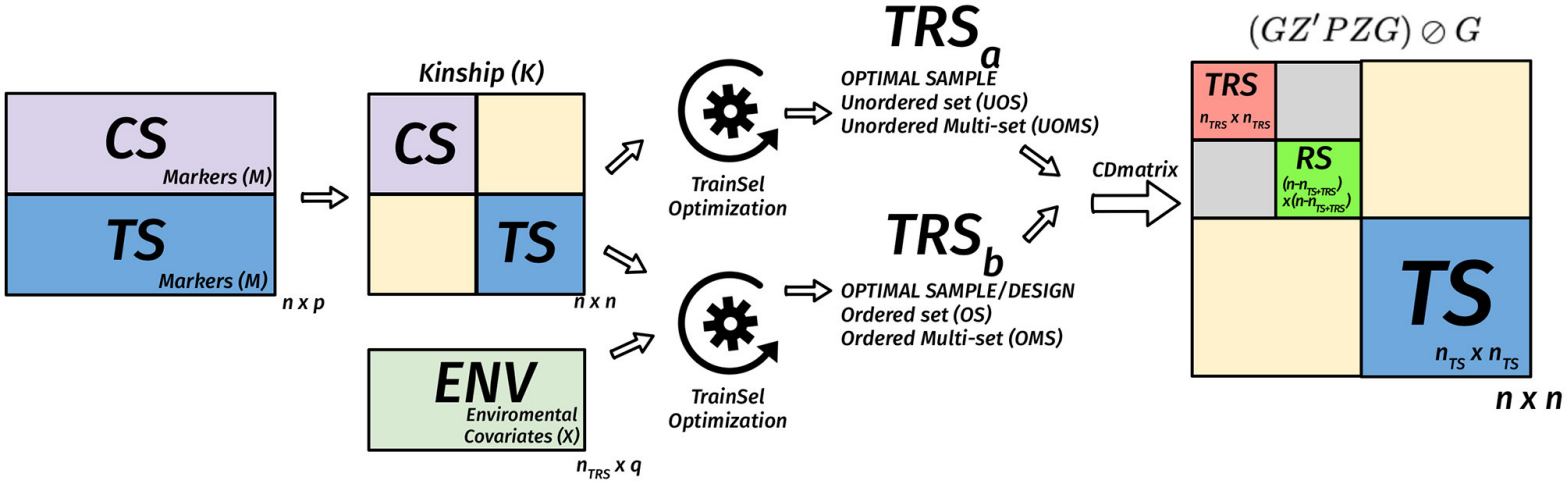
Diagram of the matrix of the CD criterion in the TRS optimization for unordered and ordered experiments for the targeted case. We assume that we have *n*_*TS*_ genotypes in the TS, *n* − *n*_*TS*_ genotypes in the CS, *n*_*TRS*_ genotypes are selected to the TRS from the CS. In this case, the *n* × *n* kinship matrix is used to calculate the *n* × *n* CD matrix for a given TRS, then the mean CD is calculated based on certain diagonals that correspond to TS. The optimization algorithm is used to find the best TRS. When provided with a design matrix that has heterogeneous rows, then we are also looking for a design in addition to the selection of a TRS. In this case, both the kinship matrix and the *n*_*TRS*_ × *q* environmental covariates matrix are used to calculate the *n* × *n* CD matrix for a given TRS, then the mean CD is calculated based on diagonals that correspond to TS. The optimization algorithm is used to find the best TRS and best design with this TRS.

**Figure 4:**
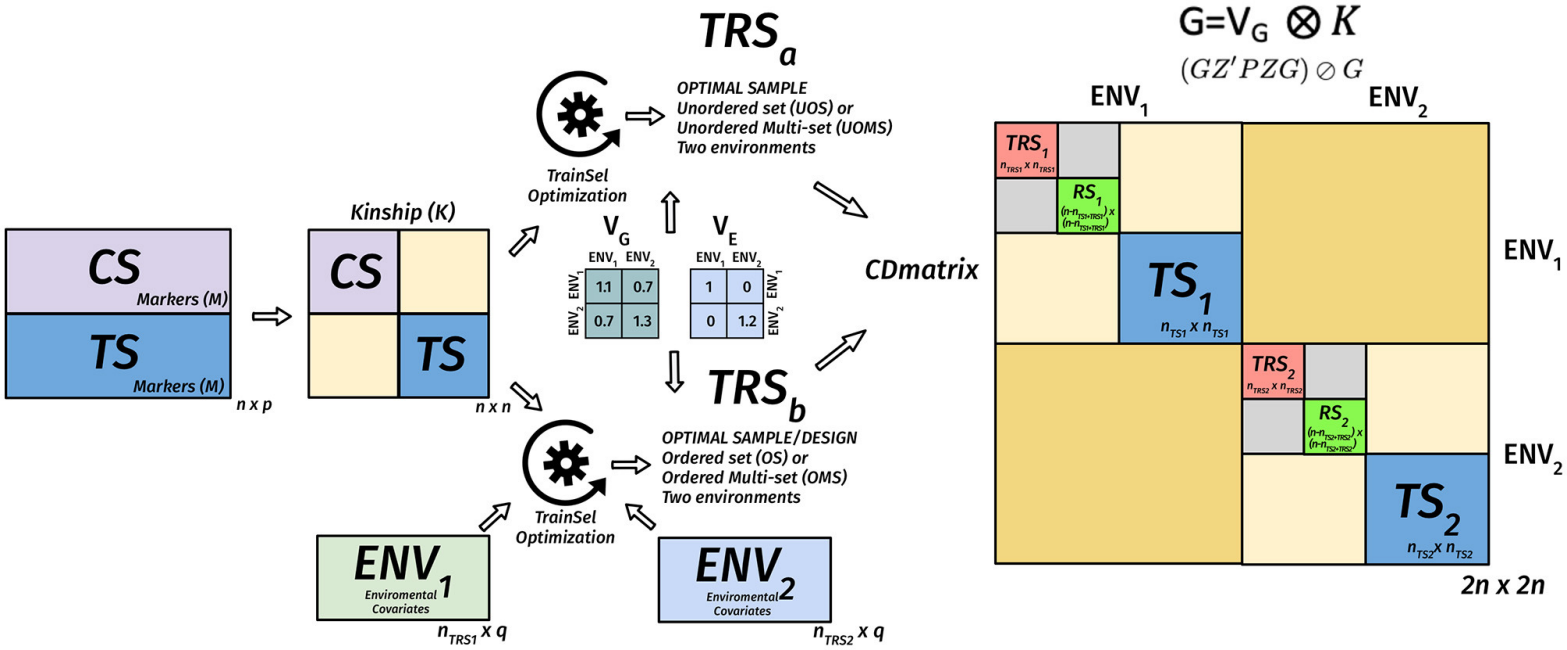
Diagram of the matrix of the CD criterion in the TRS optimization for unordered and ordered experiments for the targeted case in a multi-environmental phenotypic experiment. We assume that we have *n* genotypes, *n*_*ts*_ of them are in the target set (TS), the remaining of in the candidate set, *n*_*TR*_*S*__1__ genotypes are selected to the TRS in environment 1, *n*_*TR*_*S*__2__ genotypes are selected to the TRS in environment 2. Two environments are assumed to have a positive genetic covariance, and this is expressed in *V*_*k*_. The residual genetic covariance expressed in VE is diagonal, meaning that errors are uncorrelated between the two environments. These covariance matrices along with the genomic relationship matrix and if provided environmental covariates matrices for the environments are used to calculate the CD matrix (2*n* × 2*n*) for a given design. The mean of the diagonals of this matrix that correspond to the TS is used as a criterion for evaluating different designs. The optimization algorithm tries to find the design that maximizes this criterion.

**Figure 5:**
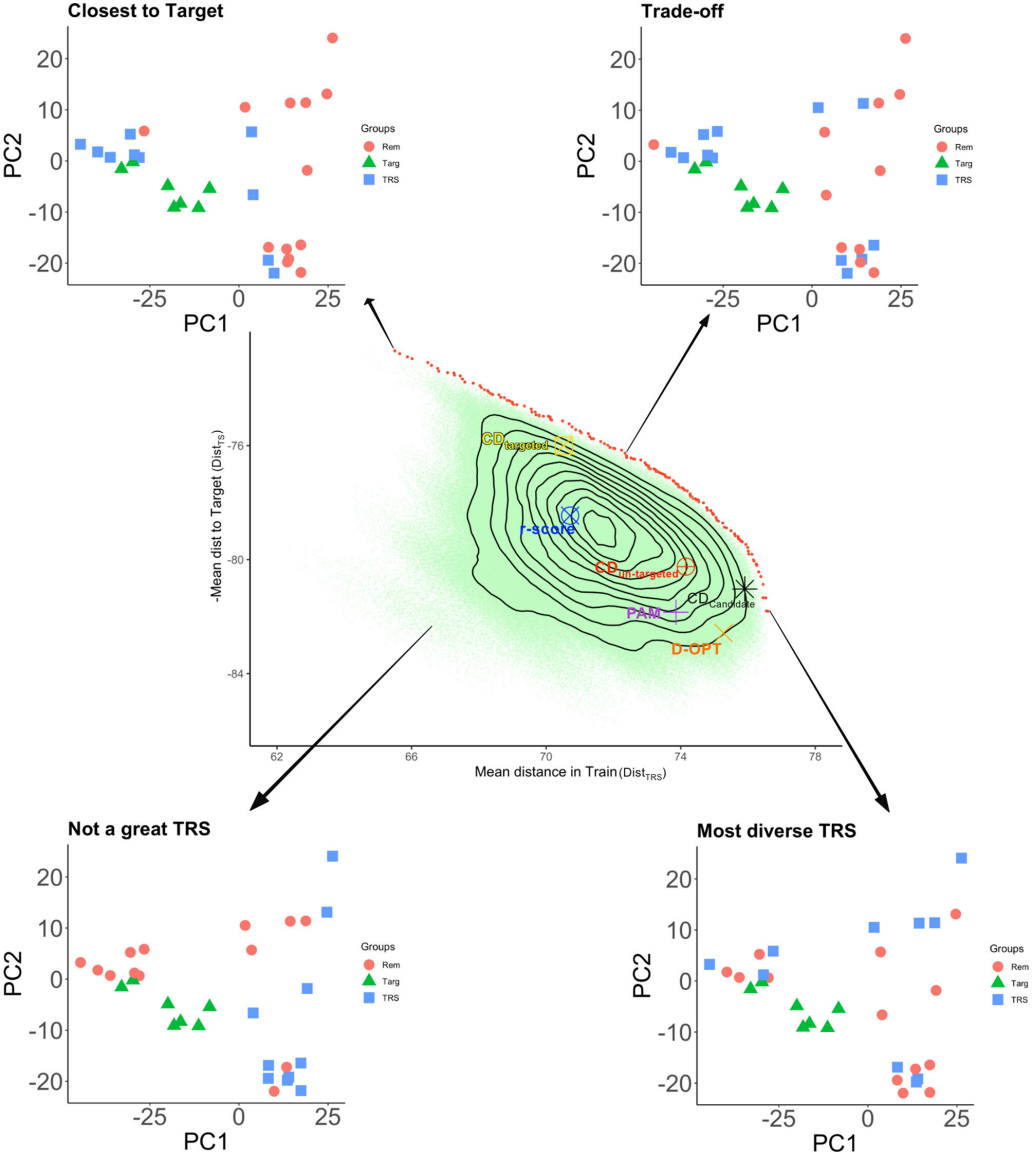
Pareto front for minimizing the mean genomic distance of TRS genotypes to the TS genotypes (i.e., maximizing the negative of this quantity), and maximizing the mean genomic distance among the TRS genotypes. The figure represents a toy example where a sample of size 10 is selected from 23 candidate genotypes to predict 7 TS genotypes (the total number of different solutions is 23 choose 10 which is more than a million). Target genotypes along with selected TRS and the remaining sets are displayed in the genotypic space represented by the first two principal components of the marker matrix. With different symbols and colors, we indicate the optimal CD TRS's for targeted and un-targeted cases, D-optimal TRS, and the TRS selected by PAM. The red dots are the TRS that are on the Pareto front, i.e., no other TRS will be better than any of these for both criteria (non-dominated solutions). All the brown dots are dominated by the same two criteria. We get the most diverse set when the mean genetic distance in the TRS is maximal. We get a TRS closest to the TS when we minimize the mean genetic distance (maximize the negative) of TRS to TS. All of the parametric design criteria and PAM are dominated. Among those, CDmean targeted gives a TRS that is close to the TS. The remaining optimal TRS's are genetically diverse. The most genetically diverse set among the optimization criteria is the CDmean calculated for all genotypes in CS.

- Parametric design criteria are based on the assumption that the experimenter has specified a model before collecting the training data. These criteria usually depend on a scalar function of the information matrix for the model parameters which indicates the sampling variances and covariances of the estimated parameters or inferences of the model made from these models such as predictions for new individuals. Many popular designs such as the *A*−, *D*−, *E*− criteria (Kiefer et al., 1985) are derived using a linear model as the underlying model. A linear model is a regression model where a response variable is modeled as a linear function of features that are functions of the explanatory variables plus some residual error:
  y=Xβ+ϵ
  where ***y*** is the *n* dimensional vector for independent realizations of the response variable, ***X*** is the *n* × *p* design matrix for the corresponding explanatory variables and ***X*** is the *n* × *q* feature matrix, ***ϵ*** is the *n* dimensional vector of independent residual terms which we assume to have mean zero and fixed variance $\sigma_{e}^{2}$ and finally, ***β*** is the *q* dimensional vector of regression coefficients. The least-squares estimator for the regression coefficients is given by $\hat{\beta }={\left(X^{\prime }X\right)}^{-1}X^{\prime }y$ and for this estimator of the coefficients we can write the variance-covariance matrix as
  $$Cov\left(\hat{\beta }\right)=\sigma_{\epsilon }^{2}\left({\left(X^{\prime }X\right)}^{-1}\right).$$
  Now, suppose we have a certain design we want to evaluate which is expressed in a specific design matrix ***X***_*TRS*_. Since we can write the covariance of the estimated coefficients as ${\left(X_{TRS}^{\prime }X_{TRS}\right)}^{-1}$ up to a proportionality constant (which is the same for all other possible designs), we can use a function of this matrix to compare it with other designs. In general, a scalar function of this matrix is used to order the different designs. D-optimality criterion, for instance, can be expressed as $\left|\left(X_{TRS}^{\prime }X_{TRS}\right)\right|$, and designs with higher values are considered better. A-optimality criterion is expressed as $trace\left[(X_{TRS})\prime X_{TRS}{)}^{-1}\right]$, and designs with lower values are considered better.
  Some other criteria such as *CDmean*, *PEVmean*, (Laloë, 1993; Rincent et al., 2012; Isidro et al., 2015) rely on a mixed model as the underlying model: In the linear mixed-effects model of interest, the observations are assumed to result from a hierarchical linear model:
  $$y=E\beta_{env}+Zu+\epsilon$$
  with ***E*** is the *n* × *p* design matrix for the environmental covariates, ***β***_*env*_ is the *p* vector of the effects of the environmental covariates, ***Z*** is the *n* × *N* design matrix for the *N* genotypes in the candidate set, ***ϵ*** ~ *N*_*n*_(**0**, ***R***) is independent of ***u*** ~ *N*_*q*_(**0**; ***G***). When using this mixed model in genomic prediction for a single environment, we use $G=\sigma_{k}^{2}K$ and $R=\sigma_{e}^{2}I$, where ***K*** is the relationship matrix of the genotypes (CS and if available the TS). When we use this mixed model with a multi-environmental genomic prediction, we assume ***G*** = ***V***_*k*_ ⊗ ***K*** and *R* = ***V***_*e*_ ⊗ ***I***.
  For this model, the CD matrix of $\hat{u}$ for predicting ***u*** is given by
  $$\left(GZ^{\prime }PZG\right)⊘G$$
  where ***P*** = ***V***^−1^ − ***V***^−1^***E***(***E***′***V***^−1^***E***)^−1^***E***′***V***^−1^ is the projection matrix and ⊘ expresses the element-wise division. Usually, the mean of certain diagonal elements of the CD matrix is used to measure the quality of a sample. For instance, in a targeted design, the mean of the diagonal elements that correspond to the TS genotypes are used. When the design is un-targeted, we can use the mean over the diagonals that correspond to the remaining set. Another approach involves the calculation of the CD matrix for a given set of contrasts then taking the mean of the diagonals of this matrix (Rincent et al., 2012, 2017). In Figures 2–4, we diagrammatically illustrate the different populations, input matrices, the different parts of the CD matrix, and the process of optimization.
- Non-parametric designs criteria are model-free, i.e., they do not rely on models we intend to use with the resulting data. Some nonparametric designs are based on distance or similarity measures and aim to spread the TRS over the design space (space-filling design). Different measures or metrics quantify how a set of points is spread out. Some examples are: (i) partition around medoids (PAM) where the objective is to find a sequence of objects called medoids that are centrally located in clusters for a given distance measure, (ii) the maximin criteria are such that the minimum distance among the TRS is maximized, (iii) the minimax design (Johnson et al., 1990) where the TRS is such that the maximum of the minimum distances from the TRS to the rest of the CS or the TS is minimized, (iv) the Latin hypercube sampling divides the design region evenly into cubes and ensuring that the sample contains just one point in each such segment and aims at ensuring that each of the scalar inputs has the whole of its range well scanned, according to a probability distribution, and (v) the minimum spanning tree (MST) (Dussert et al., 1986). An MST is a tree that connects all the candidate design points and whose total edge lengths are minimal. Once a spanning tree of the candidate points is built, the mean and SD of edge lengths can be calculated. The spanning trees with the smallest mean are called minimal and among them, the ones with high variance are preferred. A TRS from an MST can be obtained by recursively pruning out, from the candidate set, the candidate points on the leaves of the MST with small edge lengths (Guo et al., 2019).
  Non-parametric designs such as space-filling designs are well suited to the initial exploration objective. They can be used to select a smaller candidate set from a bigger candidate set to reduce the computational complexity of optimizing parametric design criteria.
- **Multiple design criteria**. Multiple models optimal experimental design criteria try to overcome the choice issue by combining more than one criteria into one *via* some type of averaging on multiple-objective optimization methods (Pukelsheim, 1993; Akdemir and Sánchez, 2016). In this approach, the Pareto front approach is used to evaluate several criteria. The Pareto front is a set of non-dominated designs, i.e., as compared to the design points on the frontier, no other design point can be found that does not degrade at least one of these criteria values (as shown in Figure 5).

Many GS experiments will be performed in several environments and then the TRS optimization aims to find subsets of genotypes from the candidate set to be tested in each of the environments and perhaps the corresponding designs within some of these environments to address the heterogeneity within environments. The use of CD for this situation is illustrated in a diagram in Figure 4.

## 4. TRS Optimization for Sparse Phenotyping

The most important current bottleneck in plant breeding programs is the phenotypic evaluation (Crossa et al., 2017). Although genotyping is still costly, next-generation sequencing has decreased genotyping cost more than 100K folds in the last 20 years (National Human Genome Research Institute, 2020), and therefore, phenotyping needs to be optimized within a breeding program. The use of GS in breeding programs is potentially costly without the careful design of populations. When designing the implementation of the GS scheme into the breeding cycle, breeders need to focus first on several aspects: (i) to generate a specific breeding database for GS, (ii) to choose the filial generation to start GS, and (iii) to select the TRS to start GS modeling (Albrecht et al., 2011; Clark et al., 2012). The design of the TRS, also called optimization of the TRS, is the breeding process that uses the information from these aspects to create a TRS to start the GS process.

Training set optimization consists of choosing (within a panel of candidates) a set of training individuals that will better predict un-phenotyped germplasm in a TS. TRS optimization has attracted notable interest in the breeding community for several reasons (Table 1). First, the fact that predictions are based on markers or line effects calculated on the TRS raises the question of how to select the TRS to increase the efficiency and effectiveness of GS. Second, currently, the high cost of phenotyping makes the phenotype information the most important constraint in plant breeding programs. Better allocation of resources within plant breeding programs by observing a small size but representative TRS would reduce phenotypic cost and increase the quality of the phenotypic data by focusing on more expensive traits with more sophisticated instruments, or increasing complementary measurements of the same traits (sparse or selective phenotyping). Third, the traditional optimization process based on random sampling as a strategy to create the TRS does not always lead to an increase in predictive ability due to the under or over-representation of the genetic information in the TRS. The TRS optimization aims to enhance the process of sparse phenotyping, to reduce the cost of phenotyping while maintaining high prediction accuracy models.

**Table 1:** Key relevant scientific studies on training set (TRS) optimization.

**Study**	**CDmean**	**PEVmean**	**Clustering**	**Other criteria**	**Package**
Rincent et al. (2012)	✗	✗	–	–	Own code
Isidro et al. (2015)	✗	✗	✗	–	Own code
Akdemir et al. (2015)	✗	✗	✗	–	**STPGA**
Lorenz and Smith (2015)	–	–	–	Levels of TRS relationship	Own code
Bustos-Korts et al. (2016)	✗	–	✗	Uniform Sampling	Own code
He et al. (2016)	–	–	–	Random	–
Rincent et al. (2017)	✗	–	✗	CDpop and Crit_Kin	Own code
Neyhart et al. (2017)	✗	✗	–	Top and bottom proportion	Own code
Cericola et al. (2017)	–	–	–	Random sampling	Own code
Momen and Morota (2018)	✗	✗		Additive and Non-additive	Own code
Norman et al. (2018)	–	–	✗	Random	Own code
Akdemir and Isidro-Sánchez (2019)	✗	✗	–	D and A-OPT	**STPGA**
Ou and Liao (2019)	✗	✗	✗	r-score	**TSDFGS**
Mangin et al. (2019)	✗	✗	–	EthAcc	Own code
Guo et al. (2019)	✗	✗	PAM	FURS	**STPGA**
de Bem Oliveira et al. (2020)	–	–	–	Random, Family Random	Own code
Adeyemo et al. (2020)	–	–	✗	–	Own code
Mendonça and Fritsche-Neto (2020)	–	✗		–	**STPGA**
Olatoye et al. (2020)	✗	–	–	Random	Own code
Roth et al. (2020)	–	✗		Maximum and Mean relationship	**STPGA**
Sarinelli et al. (2019)	–	✗	✗	–	Own code
Tayeh et al. (2015)	✗	–	–	–	Own code
Atanda et al. (2021)	✗	–	–	Avg_GRM	Own code
Yu et al. (2020)	–	–	–	Upper Bound reliability	Own code
Ben-Sadoun et al. (2020)	✗	–	–	CDmean-multi	Own code
Heslot and Feoktistov (2020)		–	–	PEVridge	Own code
Akdemir et al. (2021)	✗	✗	✗	–	**TrainSel**
Kadam et al. (2021)	✗	✗	–	–	**STPGA**

*CDmean, Mean of the coefficient of determination; PEVmean, Mean of the predictor error variance. A cross in the cell indicates that the criterion has been used for TRS optimization. Criteria different than CD, PEV, and Clustering are shown in the column Other Criteria. The software using R is specified in the Package column*.

Two important aspects within the TRS optimization are the fact that the TRS is a dynamic populations that must be updated through the breeding cycle program, and also that the TS needs to be into account when building the TRS (Akdemir et al., 2015).

The design of the TRS was initially started in animal breeding (Habier et al., 2007, 2010; Clark et al., 2012; Pszczola et al., 2012). These studies and others in plants (Windhausen et al., 2012; Wientjes et al., 2013) were focused on the importance of the relatives for the makeup of the TRS and on how to update the TRS to improve genomic prediction across generations. They highlighted how the TRS should be composed in terms of resemblance between TRS and TS, but they did not perform any optimization process, TRS was selected randomly. A random sampling of genotypes from a CS is a risky procedure because could lead to low-quality coverage of the total genetic space especially when the CS contains population structure (Windhausen et al., 2012; Isidro et al., 2015; Bustos-Korts et al., 2016). In the last decade, many studies (Table 1) examined the importance of optimization of the TRS by comparing specific selection criteria to random sampling.

The first study highlighting the importance of using statistical approaches to develop an optimal TRS was shown by Rincent et al. (2012) (Table 1). In this study, the objective was to define which individuals from a calibration (candidate) set are the optimal ones to predict a selection (TS) candidates. The idea was to use a criterion that could minimize genetic similarity within the TRS, because of the more similar the individuals within the TRS, the more duplication of the alleles, and therefore, more redundancy. Based on concepts from the mixed model equations introduced by Laloë (1993), Rincent et al. (2012) introduced criteria that aimed to maximize the reliability CD, the square correlation between GEBVs and true breeding values or minimized the prediction error variance (PEV) on the CS. In this study, they used a generalized version of CD and PEV (the contrast between breeding values). They showed that the optimization criteria improved prediction accuracy when comparing with random sampling. Rincent et al. (2012) have shown that mean of the coefficient of determination (CDmean) captured more genetic variability when building the TRS than mean of the prediction error variance (PEVmean) and that an optimized set of 100 lines achieved on average the same prediction accuracy as a set of 200 lines selected at random.

Isidro et al. (2015) proposed stratified sampling and stratified CD as alternative algorithms to improve the optimization of TRS under population structure effects. The optimization of the TRS based on genomic relationships resulted in higher prediction accuracies when compared with random sampling. In this study, they concluded that the optimization of the TRS depended on the interaction of trait architecture and population structure and on the ability of the algorithm to capture phenotypic variance. In the same year, Akdemir et al. (2015) derived a computationally efficient approximation to the PEV based on principal components of the genotypes as a criterion for TRS design that showed less computational burden than previous criteria. These studies were the first ones that open the door to other strategies to optimize the TRS. Bustos-Korts et al. (2016) proposed a TRS construction method that uniformly sampled the genetic space comprised by the target population (TS) of genotypes, although, the results were similar to CDmean.

Other studies also stressed the importance of considering an other way to construct the TRS by random sampling (Lorenz and Smith, 2015; He et al., 2016; Cericola et al., 2017; Neyhart et al., 2017; Norman et al., 2018; de Bem Oliveira et al., 2020; Olatoye et al., 2020), clustering approaches (Akdemir et al., 2015; Isidro et al., 2015; Bustos-Korts et al., 2016; Rincent et al., 2017; Norman et al., 2018; Guo et al., 2019; Sarinelli et al., 2019; Adeyemo et al., 2020), by using different levels of relatedness between TRS and TS (Lorenz and Smith, 2015; Berro et al., 2019; Roth et al., 2020) or by using other alternatives algorithms to CD-mean and PEV-mean such as different design matrix algorithm (Akdemir and Isidro-Sánchez, 2019), estimated theoretical accuracy (EthAcc) (Mangin et al., 2019), upper bound reliability (Yu et al., 2020), or the Fast and Unique Representative Subset Selection (FURS) (Guo et al., 2014). A criterion that is derived directly from Pearson's correlation between GEBVs and phenotypic values of the TS derived from the GBLUP model showed higher predictive ability than CD and PEV (Ou and Liao, 2019). Most aforementioned approaches above, do not use information from the TS while building the TRS, which is detrimental for prediction accuracy (Lorenz and Smith, 2015; Akdemir and Isidro-Sánchez, 2019; Ou and Liao, 2019). The main reason for the decrease in accuracies is because the most informative TRS to predict the TS is the one where individuals are more closely related to the TS. This is because when pairs of individuals are closely related, they tend to inherit QTL blocks in the same linkage phase (Andreescu et al., 2007; Habier et al., 2010). This is especially critical when there is low marker density coverage because the assumption in GS of getting at least one marker in QTL with the trait of interest will not be perfectly met. The genetic relatedness between TRS and TS was addressed by Lorenz and Smith (2015), Rincent et al. (2017), and Akdemir and Isidro-Sánchez (2019). Recently, Atanda et al. (2021) used the average genomic relationship (*Avg*_*G*_*RM* in Table 1) between a specific line in the TRS and all lines in the TS, and they statistically significant increase in the accuracies when compared with CD in some bi-parental populations. Nevertheless, this approach as in Rincent et al. (2017) did not consider the possible alleles duplication within the TRS.

Training optimization selection also has been used for pre-breeding discovery. Tanaka and Iwata (2018) proposed a strategy that used genomic prediction in pre-breeding for discovering the best genotypes from a large number of candidates. They demonstrated by simulation that their Bayesian optimization could reduce the number of phenotyped accessions needed to find the best accession among a large number of candidates. Their strategy was based on predict uncertainty of the prediction rather than based only on high predicted values. Following this strategy, Tsai et al. (2021) used an augmented expected improvement for sequential phenotyping to identify the best individual from the CS. It is important to note that these studies are not focusing on building a TRS for GP, but on identifying the best candidate to be used for commercial or mating purposes. These approaches could be used when phenotyping is very expensive and not very time-consuming.

In the area of hybrid breeding, the optimization of the TRS is even more critical than in other breeding systems, since the selection of superior F1 hybrids (single crosses between fully inbred lines) implies developing first inbred lines and then identifying the best hybrid combinations between them. To facilitate this process, breeders typically split germplasm into complementary heterotic groups and select lines within each group for their ability to produce good hybrids when crossed to lines from a complementary group. The fullest assessment of single-cross performances would be a complete factorial mating design achieved by making all possible single crosses. However, the high number of lines to be evaluated per heterotic group makes this approach prohibitive (i.e., for 1,000 lines in each heterotic group, there would be 1 million possible crosses). Genomic models have been applied to hybrid prediction mainly in maize (Bernardo, 1994; Schrag et al., 2009; Technow et al., 2014; Kadam et al., 2016; Marulanda et al., 2016; Fristche-Neto et al., 2018; Seye et al., 2020), and wheat (Zhao et al., 2013, 2014, 2015; Longin et al., 2015; Marulanda et al., 2016; Schulthess et al., 2017), and less in other species such as rye (Wang et al., 2014) or sunflower (Reif et al., 2013; Mangin et al., 2017; Dimitrijevic and Horn, 2018; Heslot and Feoktistov, 2020). These studies have emphasized the interest in using TRS optimization compared to the traditional crossing designs.

In general, most of the TRS studies have used model-based parametric criteria (CDmean, PEVmean, and r-score), followed by non-parametric (i.e., PAM, FURS), and just a few studies used their own criteria (i.e., AvgGRM, U score) (Table 1). All these studies show that there is not a universal criterion to create a TRS. It will mainly depend on linkage disequilibrium between markers on TRS vs. TS, the relationship between TRS and TS (Habier et al., 2007; Goddard, 2009), the genetic architecture of the trait (McClellan et al., 2007; Jannink, 2010; Burstin et al., 2015), trait heritability (Hayes et al., 2009), and population structure effects (Isidro et al., 2015; Rincent et al., 2017).

To shed some light on the different TRS optimization criteria, we constructed a toy example where we compared several design criteria (CD, PAM, D-OPT, and r.score) with each other (Figure 5). In this example, there were 30 genotypes in total, seven of these genotypes were selected as the TS. The remaining 23 genotypes were used as the CS. We set the TRS size to 10, giving 23 choose 10 (1144066) different TRS possibilities. For each of these designs, we calculated the value of the mean genetic distance among the TRS (*Dist*_*TRS*_), and the negative of the mean genomic distance from TRS to the TS (*Dist*_*TS*_). In the Figure, the red dots are the TRS that are on the Pareto front, i.e., no other TRS will be better than any of these for both criteria (non-dominated solutions). Balancing the *Dist*_*TRS*_ and *Dist*_*TS*_ in the Pareto front gives you different TRS. For instance, when we minimize the mean genetic distance (maximize the negative) of TRS to TS, we obtained a TRS closest to the TS (top left graph). We get the most diverse TRS when the *Dist*_*TRS*_ in the TRS is maximal (bottom right graph). If you balance both distances, then we get a TRS where there is a trade-off between *Dist*_*TRS*_ and *Dist*_*TS*_. The remaining TRS on the same plot is dominated with respect to the same two criteria. A TRS is dominated if we can find another TRS that improves at least one of these criteria without deteriorating the other criterion value. All of the design criteria and PAM are dominated with respect to *Dist*_*TRS*_ and *Dist*_*TS*_. Among those, CDmean targeted gives a TRS that is close to the TS, where CDmean calculated over the candidate set (CDMEAN-Cand) comes very close to the most diverse design. The contours of the density of *Dist*_*TRS*_ and *Dist*_*TS*_ over 1144066 different TRS possibilities show that a random design on average would be dominated by all of the optimal samples and would fall far away from the Pareto frontier. It is important to understand the different trade-offs involved in choosing a good TRS since this will help the experimenter to choose a suitable TRS or a TRS selection criterion among the alternatives.

Breeding programs usually deal CS's with 1,000's or 10,000's of genotypes. Although direct enumeration of all the possible TRS's is not possible in these cases, multi-objective optimization techniques can be utilized to approximate the frontier curves and single-objective optimization tools can be used to find optimal TRS's according to several single criteria. Then a plot similar to the one presented in Figure 5 can be produced to evaluate the trade-offs among different designs. When the number of genotypes in the CS is so large that computationally intensive methods are prohibitive, we recommend using a less intensive method such as PAM or stratified sampling (Isidro et al., 2015; Guo et al., 2019), or one of the space-filling designs to reduce the number of CS to a manageable size ahead of comprehensive analysis. A practical overview of the statistical analysis needed to optimize the TRS using R and issues associated with the analysis have been addressed along with the R code in the study by Isidro y Sánchez et al. (2022). In addition, extra information can be found in the extensive vignette (https://github.com/TheRocinante-lab/TrainSel/blob/main/inst/TrainSelUsage.pdf).

## 5. Software Tools for TRS Optimization

While the practical use of TRS optimization in GS is supported by the literature, as shown above, the number of software tools for implementation is limited. As far as we are concerned, just three software have been developed and available for public use. The package STPGA Akdemir (2017) is an R package that uses a modified GA for solving subset selection problems but also allows users to chose from many predefined or user-defined criteria. Similarly, the package TSDFGS Ou and Liao (2019) is an R package that focuses on optimization of the TRS by a genetic algorithm (GA) and can be used for TRS optimization based on three built-in design criteria [CDscore, PEVscore, and Pearson correlation (r-score)]. Recently, Akdemir et al. (2021) designed a new package called TrainSel to provide many more options than previous software. For example, TrainSel can select multiple sets from multiple candidate sets, users can specify whether or not the resulting set needs to be ordered, or the power to perform multi-objective optimization. In addition, TrainSel can be used for searching for solutions to a variety of TRS and experimental design problems, such as randomized complete block design, and lattice design, etc. Furthermore, it can be also used in combinatorial optimization problems for supervised and also unsupervised learning. The strength of TrainSel is that it combines TRS optimization with a particular experimental design, which has not been implemented in both of the above alternatives by Akdemir et al. (2021).

## 6. General Guidelines for a Good TRS

In this study, we highlight some of the guidelines learned from the literature when building an optimal TRS:

- When building the first TRS is key to keep, within the TRS, the historical germplasm used to generate the breeding populations. This will allow capturing the allelic diversity within the breeding program.
- The larger the TRS size the better predictions (Daetwyler et al., 2008; Zhong et al., 2009), since most characters are quantitative with a large number of loci and a very small effect size. The number of loci affecting quantitative characters likely ranges from 2,000 to 4,000 (MacLeod et al., 2016). Although adding genetically distant individuals might decrease accuracy (Lorenz and Smith, 2015), this is not a general rule. In addition, large TRS are needed to capture rare alleles at high frequencies to obtain a reliable estimate of their effects (MacLeod et al., 2016), even for highly quantitative traits if the rare allele is present in the sequencing or the genotyping is done from coding and regulatory regions.
- Markers can capture genetic relationships among genotypes, thereby affecting the accuracies of GEBVs (Habier et al., 2007). Therefore, a genetic relationship between TRS and TS is needed to obtain high accuracies. In general, a TRS should maximize the relationship with the TS (Albrecht et al., 2011; Pszczola et al., 2012; Akdemir and Isidro-Sánchez, 2019), but should minimize the relationship within the TRS (Clark et al., 2011; Lorenz, 2013; Bustos-Korts et al., 2016; Pszczola and Calus, 2016). That is to say, if TRS and TS come from different populations or breeding generations, a drop in accuracy is expected. The main reasons for the drop in accuracy are because LD between markers and QTL, or that QTL allele frequencies and/or effects can differ among populations (Hayes et al., 2009; Wientjes et al., 2015, 2017). The difference in allele frequencies between TRS and TS can affect prediction accuracy because allele frequencies can affect the estimated genomic relationship matrix when GBLUP models are implemented.
- The TRS must be updated with new genotyped and phenotyped individuals to assure the accuracy of GEBVs, is maintained over generations. Otherwise, recombination events will decrease LD between markers and QTL (Auinger et al., 2016). As phenotypes are the current bottleneck in plant breeding programs, the quality of the phenotypes is critical to the TRS optimization.
- The design of the TRS highly depends on the TS population. For example, if your TS is highly diverse, your TRS must be built to capture that diversity, otherwise, a significant drop in accuracy might occur. That is why targeted optimization approaches are chosen when building TRS (Akdemir and Isidro-Sánchez, 2019; Akdemir et al., 2021). From Figure 5 we can observe that we get a TRS closest to the TS when we minimize the mean genetic distance (maximize the negative) of TRS to TS. Among the different TRS selection criteria, CDmean targeted gives a TRS that is close to the TS. The remaining optimal TRS's are genetically diverse but the most genetically diverse set among the optimization criteria is the CDmean calculated for all genotypes in CS. This type of evaluation of different design criteria together along with a frontier curve should shed some light on the selection of a particular TRS.
- If certain QTL with large effects for traits of interest exists, then these QTL can be given more influence while selecting the TRS. This could be done, for example in the mixed modeling framework by using the QTL as fixed effects (Spindel et al., 2016). In the non-parametric approach, more weights can be given when calculating the genetic distance matrix.
- In general, optimization criteria from mixed model theory (CDmean, PEVmean) performs better than random sampling under most scenarios, except for scenarios with a large population structure where these criteria might not be optimal (Isidro et al., 2015).

## 7. Perspectives for TRS Optimization

Genomic selection is an emergent methodology that revolutionized plant and animal breeding, by using a statistical framework that uses genome-wide markers to predict breeding values for key breeding traits. In this framework, one critical step is how to select the best individuals to train the statistical models. As shown above, there has been quite a great research in this area, but there are still some questions to be answered. Following the literature, there is no “best” strategy to optimize the TRS, and therefore, a comparison between algorithms focusing on the different factors affecting the TRS on different populations would be helpful to answers some questions regarding TRS optimization.

We envision a substantial benefit applying TRS optimization methods to hybrid prediction, and also sparse testing in multi-environment, and multi-trait experiments (Jarquín et al., 2014; Akdemir et al., 2021; Crossa et al., 2021). For instance, in hybrid prediction, TRS are traditionally constructed by methods such as top crosses, North Caroline design, etc. It has been shown that the TRS optimization methods improve hybrid prediction accuracies when comparing with the traditional design methods (Zhao et al., 2015, 2021; Fristche-Neto et al., 2018; Heslot and Feoktistov, 2020; Yu et al., 2020; Technow et al., 2021).

It is also expected that TRS selection methods will be used more commonly in multi-environmental phenotypic experiment design (Montesinos-López et al., 2019; McGowan et al., 2020) as more flexible and powerful tools such as the package R TrainSel becomes available for researchers. The use of genomic information in designing these experiments shifts the attention from replication of individuals to replication and representation of alleles in different environments.

In addition, more studies using haplotypes rather than just markers are needed, since accuracies are greater if TRS and TS share long-range haplotypes (Akdemir et al., 2015; Meuwissen et al., 2016; Scott et al., 2021). The decrease of whole genomic sequencing is allowing us to develop pan-genomes studies of many crops, which will allow us to switch from SNPs to longer more important haplotypes in the design of TRS populations. The development of haplotype-informed DNA markers will enable the selection of new haplotype combinations, which will increase the opportunity to attain optimized genetic combinations for improved performance and disrupt linkage drag (Varshney et al., 2021).

An unresolved issue in TRS optimization is the determination of the size of TRS. The size of TRS is usually dictated by the budget for the experiment, however, a breeder might need guidance for selecting a TRS size to avoid redundancy of individuals. For example, even though a breeder might have the resources to do 20 individuals, the breeder should know what is the optimal size to experiment. The optimal size of the TRS can be obtained from the multi-objective optimization framework Akdemir et al. (2019). The solutions on the Pareto front of an optimization problem Markowitz (1968), where one or more design criteria along with the TRS size are optimized, will provide the experimenter with a scenery of the optimal design space at each sample size. The usual methods of selecting a solution on a frontier can guide the determination of the TRS size.

Finally, a comparison of criteria with different populations, different genetic architectures, heritability values, and relationships among TRS and TS is needed, especially to evaluate if some previous claims in the TRS optimization area are true under the same population scenarios.

## Data Availability Statement

The datasets presented in this study can be found in online repositories. The names of the repository/repositories and accession number(s) can be found in the article/supplementary material.

## Author Contributions

JIS and DA: conception and design of the article, drafting the article, and critical revision of the article. Both authors contributed to the article and approved the submitted version.

## Funding

Results have been achieved within the framework of the first transnational joint call for research projects in the SusCrop ERA-Net Cofound on Sustainable Crop production, with funding from the Department of Agriculture, Food and the Marine grant no. 2017EN104. This project has also received funding from the European Union's Horizon 2020 research and innovation program under grant agreement No 818144. JIS was supported by the Beatriz Galindo Program (BEAGAL18/00115) from the Ministerio de Educación y Formación Profesional of Spain and the Severo Ochoa Program for Centres of Excellence in R&D from the Agencia Estatal de Investigación of Spain, grant SEV-2016-0672 (2017-2021) to the CBGP.

## Conflict of Interest

The authors declare that the research was conducted in the absence of any commercial or financial relationships that could be construed as a potential conflict of interest.

## Publisher's Note

All claims expressed in this article are solely those of the authors and do not necessarily represent those of their affiliated organizations, or those of the publisher, the editors and the reviewers. Any product that may be evaluated in this article, or claim that may be made by its manufacturer, is not guaranteed or endorsed by the publisher.
